# A Case–Control Study on the Effectiveness of Tick-Borne Encephalitis Vaccination Against Hospitalizations in an Endemic Area in Northeastern Italy

**DOI:** 10.3390/vaccines14020164

**Published:** 2026-02-10

**Authors:** Francesca Valent, Giulia Degani

**Affiliations:** SOC Igiene e Sanità Pubblica, Azienda Sanitaria Universitaria Friuli Centrale, 33100 Udine, Italy; giulia.degani@asufc.sanita.fvg.it

**Keywords:** tick-borne encephalitis, vaccination, vaccine effectiveness, case–control study, Italy, Friuli Venezia Giulia, epidemiology

## Abstract

**Background**: Tick-borne encephalitis (TBE) is a severe viral infection of the central nervous system transmitted by tick bites. Vaccination represents the only effective preventive measure, yet data on TBE vaccine effectiveness in Italy are lacking. This study aimed to evaluate TBE vaccine effectiveness and vaccination coverage in the province of Udine, an endemic area in the Friuli Venezia Giulia region of Northern Italy. **Methods**: We conducted a case–control study using linked anonymized health databases of the region, including vaccination, laboratory, and hospital admission records from 2017 to 2025. Cases were defined as residents hospitalized with a diagnosis of TBE (ICD-9-CM 063.x or 321.2) and a positive anti-TBE IgM result in serum or cerebrospinal fluid. Controls were residents tested for anti-TBE IgM during the same period but not hospitalized for TBE. Vaccination history was retrieved from the regional vaccination registry. Vaccine effectiveness was estimated through logistic regression models comparing vaccinated and unvaccinated individuals. **Results**: Between 2017 and 2025, 21 confirmed TBE hospitalizations were recorded (mean annual incidence: 0.45/100,000 inhabitants). The mean hospital stay was 13.8. Among 6065 individuals tested for anti-TBE IgM, 95.2% of cases and 81.8% of controls were unvaccinated. The estimated odds ratio of TBE hospitalization for individuals with ≥3 vaccine doses versus unvaccinated was 0.11 (95% CI: 0.02–0.88). Vaccination coverage in 2025 reached about 10% of the provincial population, with markedly higher coverage (up to 34%) in mountain districts compared with lowland areas (<5%). **Conclusions**: Although limited by small sample size, this study provides the first real-world evidence of TBE vaccine effectiveness in an Italian endemic area. Vaccination is an effective preventive measure. Given the regional epidemiology and expected increase in tick activity due to climate change, strengthening vaccination uptake and public awareness in endemic districts is strongly recommended.

## 1. Introduction

Tick-borne encephalitis (TBE) is a rare but potentially very serious disease affecting the brain and spine, caused by a viral infection transmitted by a tick bite. Most cases occur during periods of high tick activity, which in Europe extend mainly from April to November. TBE is more common in adults than in children. Clinical manifestations range from mild to severe, initially including flu-like symptoms such as fever and headache, whereas meningitis and other central nervous system involvement symptoms may develop in later stages. TBE can also result in long-lasting disability and death. Since clinical features are nonspecific, diagnosis must be confirmed by microbiological findings, in particular by the detection of specific IgM and IgG antibodies in serum and/or cerebrospinal fluid. There is no specific antiviral treatment for TBE; therefore, vaccination represents the primary strategy to prevent the disease. For this reason, vaccination is recommended for individuals living in TBE-endemic areas [1,2].

The World Health Organization (WHO) recommends that vaccination be offered to all age groups, including children, in areas where the disease is highly endemic, where the average prevaccination incidence of clinical disease is ≥5 cases/100,000 population per year (PPY), but remarks that the inclusion of vaccination against TBE into immunization programs at regional level or national level should be considered, depending on the epidemiological situation [3].

Halsby et al. reported the incidence of TBE in 34 European countries from publicly available data and estimated incidence among unvaccinated populations in 21 of them with sufficient data [4]. They reported that the incidence in 2020–2023 was ≥5/100,000 PPY in Lithuania, Latvia, Estonia, Czechia, and Slovenia and, when calculating the unvaccinated incidence, also in Sweden and Austria. Italy was identified as a country with endemic areas; however, insufficient data were available for estimating unvaccinated incidence. Nonetheless, the northeastern part of Italy borders Austrian and Slovenian regions with high endemicity [4].

**Figure 1:**
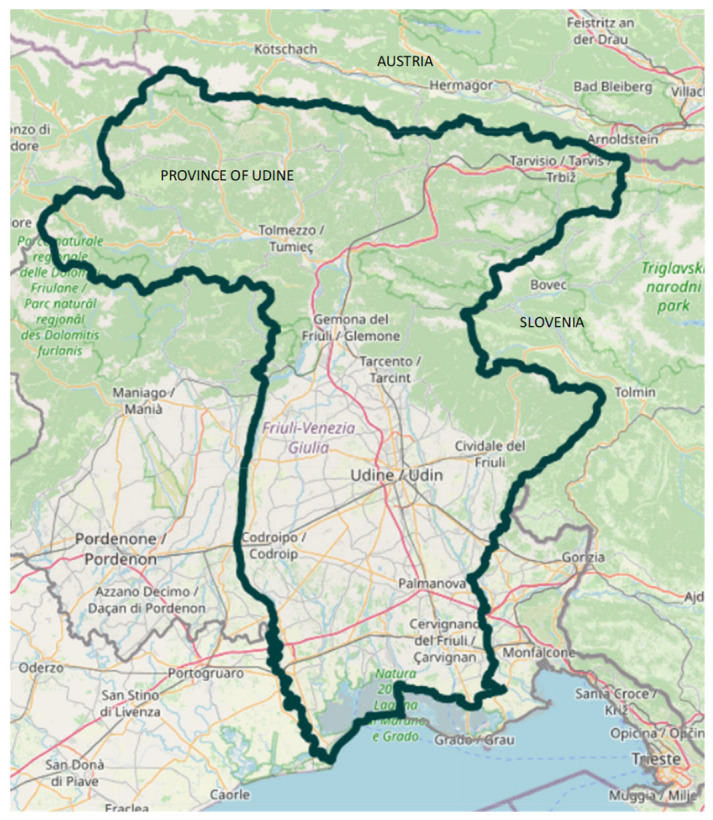
Map of the province of Udine. Green color identifies the hilly/mountainous areas.

The Friuli Venezia Giulia region, the Italian area corresponding to the former province of Udine and comprising approximately 520,000 inhabitants, includes a northern mountainous area (Gemona and Tolmezzo, along the Austrian border) and an eastern mountain–hill area (Tarcento and Cividale, along the Slovenian border) where TBE has been endemic for several years (green areas in Figure 1). The area is divided into nine Health Districts, each with a population of approximately 40–55,000 inhabitants, with the exception of the Udine district, which includes the main city (approximately 157,000 inhabitants) and the two mountain districts of Gemona and Tolmezzo, which have smaller populations (approximately 30–35,000 inhabitants). For this reason, the FVG region offered the TBE vaccine with a copayment to all residents from 2007 to 2013 and has provided it free of charge to all residents since 2013 [5,6,7]. In Italy, including Friuli Venezia Giulia, the recommended vaccination schedule consists of a three-dose primary series (at month 0, 1–3 months after the first dose, and 5–12 months after the second dose), followed by a first booster dose after 3 years and subsequent boosters according to an age-dependent schedule [8]. Panatto et al. reported that vaccination coverage in Friuli Venezia Giulia and other Italian endemic areas was suboptimal as of 2016, reaching only 5% in Friuli Venezia Giulia [9].

In Europe, vaccination against TBE is considered very effective. A systematic review including studies from five European TBE-endemic countries (Austria, the Czech Republic, Latvia, Germany, and Switzerland) reported very high effectiveness of TBE vaccination against TBE infection and/or TBE hospitalization, generally exceeding 90% across most outcomes and population groups [10].

A very large recent Swedish study, considering TBE cases and their vaccination status reported via the public health surveillance system and the vaccination history of non-cases reported from a general population survey, demonstrated very high vaccine effectiveness across all ages, ranging from 83.4% in individuals ≥ 60 years of age to 93.8% in those 16–49 years [11].

In Switzerland, a nationwide, cross-sectional surveillance study focuses on children and adolescents 0–17 years of age, comparing vaccination status among confirmed, probable, and possible TBE cases (including non-specific neurological or influenza-like symptoms in combination with appropriate serology) with vaccination status among participants in the Swiss National Vaccination Coverage Survey [12]. In this study, vaccine effectiveness (VE) for incomplete vaccination was 66.2% (95% CI: 42.3–80.2), whereas VE for complete vaccination was 90.8% (95% CI: 87.7–96.4) [12].

In Austria, which borders the north of the mountainous area of the province of Udine, a large study was recently conducted to evaluate the effectiveness of vaccination against hospitalized TBE cases. Vaccination status for cases was obtained from medical records, while data for the reference population were derived from a nationally representative household survey with weighted data. The study showed a vaccine effectiveness of 94.3% in children aged 1 to 15 years and 97.4% in adults up to 59 years of age. Irregular vaccination was also found to provide protection, although to a lesser extent than regular vaccination [13].

To our knowledge, there is no recent study assessing the effectiveness of TBE vaccination in Italy. Therefore, we conducted this study to assess whether TBE vaccination is effective against hospitalizations in the area corresponding to the province of Udine.

## 2. Material and Methods

This was a case–control study using the administrative health information system of the Friuli Venezia Giulia Region as the source of information. The health information system is a data warehouse including several health-related databases, which are anonymous but can be individually linked in a deterministic manner through an anonymous key that is randomly assigned to each individual and that is univocal in all of the databases. For this study, we linked the vaccination database, containing records of all vaccinations administered in the region; the laboratory database, containing data on all tests conducted in regional public laboratories; and the hospital admission database, including data on all hospitalizations occurring in regional hospitals.

In Italy, TBE is a notifiable infectious disease and must be reported to the Ministry of Health [14]. Notification is performed by Local Health Agencies (such as ASUFC) following physician reporting. Asymptomatic or paucisymptomatic infections are generally not notified because of the lack of clinical criteria, whereas symptomatic cases are virtually always hospitalized. Accordingly, cases in this study were defined as residents of the province of Udine who were hospitalized between 2017 and August 2025 and discharged with a diagnosis of tick-borne viral encephalitis (ICD-9-CM code 063.x) and/or viral meningitis not elsewhere classified (ICD-9-CM code 321.2) who also had a positive TBE IgM test on serum or cerebrospinal fluid either during hospitalization, or within 15 days before admission or after discharge. IgM testing in the serum and cerebrospinal fluid of TBE patients has been shown to be specific [15].

Controls were defined as all other residents of the province of Udine who underwent testing for TBE infection by identifying IgM in serum or cerebrospinal fluid during the same 9-year period. We decided not to include the non-tested population as controls, as we assumed that individuals who underwent testing were those for whom the possibility of tick bites could not be excluded and therefore they represented the actual population from which cases arose.

Data on any TBE vaccinations administered to cases and controls were abstracted from the vaccination database from 2005, when the vaccine was marketed in Italy [16]. TBE vaccinations are offered free of charge in the Friuli Venezia Giulia region, but not in most other Italian regions; therefore, it is unlikely that a resident individual was vaccinated elsewhere and not here. Since vaccination is free for residents, there is no reason to purchase the vaccine and have it administered privately outside the institutional framework. Thus, all vaccinations administered in the Friuli Venezia Giulia region and, within it, in the province of Udine are recorded in the dedicated software system and are therefore fully captured.

Hospitalized cases from the year 2017 were included because accurate laboratory data prior to that year were not available for analysis.

For both cases and controls, we assessed the number of TBE vaccine doses received before the index event (defined as hospitalization with a positive IgM test for cases, or the date of TBE IgM testing for controls), as well as the time elapsed since the last vaccine dose.

We also abstracted information on subjects’ sex, age, and area of residence, and estimated the TBE vaccination coverage. Data for 2025 are partial and include only the first 8 months of the year; nevertheless, they were included in the calculation of annual averages as the majority of TBE cases and laboratory tests occur during the summer months. For this reason, estimates of the annual average numbers of administered vaccine doses and new primary series may be slightly conservative.

### Statistical Analysis

No sample size calculation was performed for this study, as we included all subjects meeting either the case or control definitions.

We described the distribution of characteristics of cases and controls using absolute and relative frequencies for categorical variables (sex and area of residence) and mean values for continuous variables (age). Differences in age between cases and controls were assessed using the Wilcoxon’s rank sums test. *p*-values < 0.05 were considered statistically significant.

Laboratory testing rates in each district of the study area were calculated using official Istat population data for the median year of the study period (i.e., 2021).

The effect of vaccination was assessed using contingency tables, in which the association between vaccination status and outcome was tested using the two-sided chi-square test or Fisher’s exact test when expected cell counts were <5. In addition, univariable logistic regression models were fitted with case–control status as the dependent variable and vaccination status as the independent variable. Three different categorizations of vaccination status were considered: at least 2 doses of TBE vaccine vs. none; at least 3 doses vs. none; and the number of doses as a discrete continuous variable.

Multivariable logistic regression was used to estimate the effect of receiving at least 3 doses of TBE vaccine vs. none on the risk of hospitalization, adjusting for age (continuous, in years), sex, district of residence, and calendar year. Odds ratios (OR) and 95% Confidence Intervals (95%CI) were calculated for both univariable and multivariable logistic regression models.

## 3. Results

In the Italian province of Udine, since 2013, when free vaccination was offered to the entire population living in the area, an average of 11,800 vaccine doses have been administered annually (13,100 when excluding years 2020 and 2021, during which routine vaccination activities were reduced because of the SARS-CoV-2 pandemic), corresponding to an average of 3400 new vaccination cycles per year (3850 excluding years 2020 and 2021). Overall, 10% of the current population has completed at least the three-dose primary series, with substantial variability across the nine Health Districts, ranging from 4% in the two lowland districts closer to the coast to 25% and 34% in the two mountain districts of the province.

**Figure 2:**
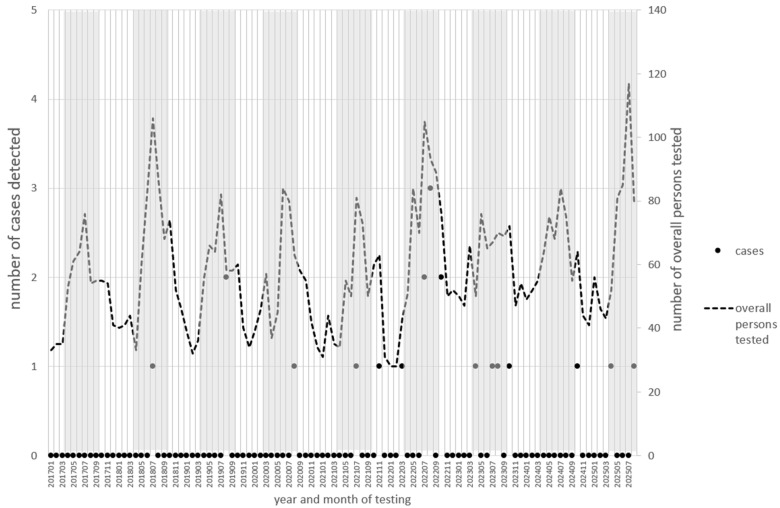
Time distribution of testing for IgM against TBE and case detection. Months from April to October are in gray bands.

From 2017 to 2025, 21 residents of the province of Udine were hospitalized for microbiologically confirmed tick-borne encephalitis, corresponding to an average annual incidence rate of 0.45 TBE hospitalizations per 100,000 inhabitants. The mean length of hospital stay was 13.8 ± 7.3 days (median 13 days, range 4–30 days) and the inpatient tariff attributed to each hospitalization was 7618 euros. During the same period, an additional 6044 individuals underwent laboratory testing for anti-TBE IgM antibodies. The temporal distribution (by year and month) of testing and case detection is shown in Figure 2. The number of individuals tested peaked during the summer months, and the majority of cases were detected in the same season.

The characteristics of the cases and controls are shown in Table 1. Hospitalized cases were, on average, older than controls (*p*-value of Wilcoxon’s rank sums test = 0.1048), with age ranging from a minimum of 33 to a maximum of 86 years. A total of 148 children and adolescents 0–15 years of age were tested, but none had positive IgM against TBE. The number of male cases was higher than the number of female cases, whereas the number of female controls was slightly higher than males, but the difference was not significant (*p*-value of chi-square test = 0.4511). From 2017 to 2025, despite the small annual number of cases, an increase appears in the proportion of cases among those who were tested (*p*-value of Fisher’s exact test = 0.0389), whereas the number of persons tested did not vary substantially. The maximum number of inhabitants tested lived in the most populous district of Udine; however, when taking into account the population living in each district, in 9 years, the testing rate was much higher in the mountain and hill areas, as follows: San Daniele, 10.7 per 1000 inhabitants; Tarcento, 16.7 per 1000; Cividale, 12.8 per 1000; Codroipo, 8.3 per 1000, Udine, 11.1 per 1000; Cervignano, 5.0 per 1000; Latisana, 5.5 per 1000; Tolmezzo, 22.0 per 1000; Gemona, 23.7 per 1000. Also, the proportion of cases was much higher in the mountain and hill areas than in the others (*p*-value of Fisher’s exact test = 0.0123). Fourteen cases (66.7%) occurred in the population of the two small mountain districts of Gemona and Tarcento (accounting for approximately 13% of the population). Moreover, 95.2% of cases were never vaccinated against TBE, vs. 81.8% of controls (*p*-value of Fisher’s exact test = 0.1547).

The OR of TBE hospitalization for subjects with ≥3 vaccine doses (primary series) vs. those not vaccinated was 0.26 (95%CI: 0.03–1.95). The results did not change significantly when we considered subjects with ≥2 vaccine doses vs. those not vaccinated (OR = 0.23; 95%CI: 0.03–1.75), and when we considered those with ≥4 vaccine doses vs. those not vaccinated (OR = 0.36; 95%CI: 0.05–2.69). After adjusting for age, sex, district of residence and calendar year, the OR of TBE hospitalization for subjects with ≥3 vaccine doses vs. those not vaccinated became 0.11 (95%CI: 0.02–0.88). Sex, calendar year, and district were not significantly associated with the likelihood of hospitalization, whereas each increasing year of age was associated with a 2% increase in the likelihood of hospitalization (OR = 1.02; 95%CI: 1.00–1.05).

The unique vaccinated case had received four vaccine doses: the full three-dose cycle 9 years before contracting the disease and the fourth dose (i.e., the first booster dose) 3 years later. Another case, who was never vaccinated before contracting the disease, started a vaccination cycle 8 months after the infection.

## 4. Discussion

This study provides an overview of the incidence of TBE infections requiring hospitalization in an endemic area of Northern Italy. As expected, the incidence is rare (less than one case per 100,000 inhabitants per year); however, since 2018, there has not been a single year without reported cases of TBE in this relatively small area (about 4900 km^2^). In fact, in 2022, as many as eight cases were recorded. The issue of TBE is also relevant because of the relatively high frequency of laboratory testing in the province of Udine: on average, almost 700 tests are performed each year, meaning that more than one person per 1000 inhabitants is tested annually, mostly during the summer. The economic impact of TBE is particularly high in hospitalized cases. Scaggiante et al. estimated that, although Italy reports fewer TBE cases than neighboring European countries, the disease can still have a significant health and economic impact in endemic areas, with hospitalization costs ranging from 5813.7 to 7352.5 euros [17]. In our hospitalizations, with an average length of stay of 13 days, the corresponding tariff was similarly high, at approximately 7600 euros.

To our knowledge, this is the first independent study attempting to estimate the effectiveness of TBE vaccination in an Italian endemic area. Given the small number of hospitalized TBE cases registered in this area, our estimate of the association between vaccination and outcome was quite imprecise. Nonetheless, the magnitude of the effect is clearly in favor of vaccination. Regardless of whether the effect is measured for completion of at least one full vaccination series (three doses), receipt of at least the first two doses, or the completion of a full series plus at least one booster, crude vaccine effectiveness against hospitalized cases can be estimated to range between 64% and 77%. When adjusting for potentially confounding variables such as age, sex, district of residence, and calendar year, vaccine effectiveness increased to 89% (95%CI: 12–98%).

To increase statistical power, we could have considered all subjects with positive TBE serological tests as cases. However, as suggested by Vilibic-Cavlek et al. [18], serological results alone should be interpreted with caution, including the possibility of cross-reactivity, especially in areas where several flaviviruses co-circulate. Thus, the decision to restrict the case definition to laboratory-confirmed hospitalized cases allowed for greater specificity and helped prevent false positive tests (not uncommon), which may not be supported by the patient’s medical presentation and history, thereby reducing the risk of selection bias in the results.

The magnitude of the protective effect of TBE vaccination observed in our study is slightly lower than the vaccine effectiveness reported in Sweden, although it is consistent with the lower 95% confidence limits of the Swedish estimates [11]. The somewhat lower effectiveness observed in our study may reflect true geographical differences in vaccine breakthrough; alternatively, it may simply be due to the smaller size of our study and the resulting imprecision of our estimates. Another possible explanation is our choice of controls, which included all individuals living in the province who underwent serological testing for TBE infection but were not hospitalized. We chose to restrict our study to individuals who underwent TBE IgM testing in an attempt to exclude the portion of the population with no exposure risk. For instance, individuals living in the southern part of the province who never visit the northern hills and mountains are virtually never exposed, are very unlikely to be tested, and are unlikely to be vaccinated. This approach was intended to select controls from the same source population that gave rise to the cases.

In addition, by enrolling only individuals who underwent testing, we aimed to reduce confounding by health-seeking behavior. However, health-seeking behavior may still vary among tested subjects, resulting in residual confounding. Moreover, conditioning the analysis on testing may have introduced a form of selection bias known as collider stratification bias, potentially distorting the association between vaccination status and TBE hospitalization. In fact, testing may be influenced by vaccination status on one hand and by the severity of infection symptoms (and hospitalization) on the other; conditioning on testing may therefore lead to the observation of a spurious association between vaccination and hospitalization [19,20]. In our study, since vaccination may have led either to reduced testing (due to the belief of being immune to TBE infection or to milder symptoms in the event of infection) or to increased testing because of greater awareness among vaccinated individuals, the direction of the potential bias introduced by conditioning on testing is unclear. Compared with Sweden, TBE vaccine coverage in our area was lower, with an average of approximately 10% of the population of the province of Udine having completed the three-dose primary vaccination series. However, vaccine uptake is currently higher than previously reported in this area [9], with more than 3000 individuals receiving the primary series each year, indicating increasing awareness among the population of both the disease and the importance of protection. As expected, vaccination coverage is substantially higher among residents of the mountain and hill districts, where the circulation of infected ticks is greatest.

One strength of our study is that, unlike the Swedish and Austrian studies whose estimates of vaccination coverage were based on a population survey and therefore subject to various potential biases [11,13], our coverage data, as well as the vaccination status of cases and controls, were not self-reported but extracted from the official vaccination database of the Health Agency of ASUFC. The database covers the entire population of the province of Udine, is objective, and has virtually 100% completeness. Consequently, although our study lacks precision, the risk of information bias is minimal.

Since there is no specific antiviral treatment for TBE, vaccination represents a key tool for disease prevention in endemic areas. Despite its imprecision, our study indicates that vaccination is highly effective in preventing serious disease in this setting; therefore, we strongly recommend vaccination for the entire population. This recommendation is further supported by Müller et al. [21], who, using Germany as a case study, showed that TBE vaccination is good value for money even in settings where incidence falls below the WHO threshold of highly endemic areas, i.e., ≥5 cases/100,000 population per year [3], especially in the population aged 60–85 years.

However, Ixodes ricinus ticks in the area of Udine and the greater Friuli Venezia Giulia region also transmit Borrelia, which is the pathogen causing Lyme disease. As of today, there is no vaccine against Lyme Borreliosis; therefore, behavioral prophylaxis (e.g., avoiding walks in tall grass, wearing light-colored clothing with long sleeves and pants, applying repellents to the skin and permethrin to clothing in the period from March to October at least) is strongly recommended for both people living in the region and for tourists who come and visit wooded areas and meadows [22].

Considering that a warming climate in the northeast of Italy will likely increase tick survival, shorten life-cycles, and lengthen the duration of tick activity seasons [23], we expect that the problem of tick bites and the infections they transmit will become even more significant in the coming years.

For this reason, vaccination should be even more strongly recommended not only for the population residing in the province of Udine, but also for national and international travelers who decide to visit these areas. In fact, Srinivasan et al. reported that, despite existing recommendations for TBE vaccination for travelers to endemic areas in Europe, cases of travel-associated TBE among international travelers continue to be reported and, in light of the expanding geographic distribution of TBE-endemic areas and the rising incidence of TBE in Europe, strengthened efforts are warranted to improve risk awareness and to promote uptake of TBE vaccination among those traveling to endemic regions [24].

## 5. Conclusions

This is the first study estimating the effectiveness of TBE vaccination in an Italian endemic area. Despite the small number of cases and the large imprecision in the results, vaccination was associated with reduced likelihood of hospitalizations due to TBE.

From a public health perspective, in addition to behavioral prophylaxis, which is always recommended in endemic areas to avoid tick bites, vaccination is an effective tool to ensure that the population is protected against TBE. It is strongly recommended to all inhabitants of the Friuli Venezia Giulia region and the province of Udine in particular, and to all those persons who, for professional reasons or in their free time, engage in outdoor activities in the mountains and hills of these areas.

## Figures and Tables

**Table 1:** Characteristics of 21 hospitalized TBE cases and 6044 controls undergoing TBE IgM tests from 2017 to 2025 in the northeastern Italian province of Udine.

	Cases	Controls
Age (mean ± standard deviation, median)	59.6 ± 13.7, 61	52.9 ± 18.5, 54
Sex (*n*, %)		
Male	12 (57.1%)	2956 (48.9%)
Female	9 (42.9%)	3088 (51.1%)
Year (*n*, %)		
2017	0 (0%)	616 (10.2%)
2018	1 (4.8%)	735 (12.2%)
2019	2 (9.5%)	625 (10.3%)
2020	1 (4.8%)	640 (10.6%)
2021	2 (9.5%)	607 (10.4%)
2022	8 (38.1%)	769 (12.7%)
2023	4 (19.0%)	733 (12.1%)
2024	1 (4.8%)	726 (12.1%)
2025 (January to August)	2 (9.5%)	593 (9.6%)
District of residence (*n*, %)		
San Daniele	2 (9.5%)	493 (8.2%)
Tarcento	1 (4.8%)	665 (11.0%)
Cividale	1 (4.8%)	633 (10.5%)
Codroipo	0 (0%)	415 (6.9%)
Udine	3 (14.3%)	1748 (28.9%)
Cervignano	0 (0%)	281 (4.6%)
Latisana	0 (0%)	281 (4.6%)
Tolmezzo	7 (33.3%)	792 (13.1%)
Gemona	7 (33.3%)	736 (12.2%)
Number of TBE vaccine doses received		
None	20 (95.2%)	4943 (81.8%)
1	0 (0%)	49 (0.8%)
2	0 (0%)	108 (1.8%)
3	0 (0%)	259 (4.3)
4	1 (4.8%)	626 (10.4%)
5	0 (0%)	52 (0.9%)
6	0 (0%)	6 (0.1%)
7	0 (0%)	1 (<0.1%)
Total	21 (100.0%)	6044 (100%)

## Data Availability

The raw data supporting the conclusions of this article will be made available by the authors on request.
